# Impact of physical exercises on immune function, bone mineral density, and quality of life in people living with HIV/AIDS: a systematic review with meta-analysis

**DOI:** 10.1186/s12879-019-3916-4

**Published:** 2019-04-24

**Authors:** Sam Chidi Ibeneme, Franklin Onyedinma Irem, Nneka Ifeyinwa Iloanusi, Amarachi Destiny Ezuma, Fortune Elochukwu Ezenwankwo, Philip Chinedu Okere, Amaka Obiageli Nnamani, Salome Nwaelom Ezeofor, Ngozi Regina Dim, Gerhard Fortwengel

**Affiliations:** 1 Department of Medical Rehabilitation, Faculty of Health Sciences, University of Nigeria, Enugu Campus, Enugu, Nigeria; 2 Department of Radiation Medicine, Faculty of Medical Sciences, College of Medicine, University of Nigeria, Ituku-Ozalla Campus, Enugu, Nigeria; 3 Exercise Immunology/Palliative care unit, Department of Physiotherapy, University of Nigeria, Teaching Hospital, Ituku/Ozalla, Enugu State, Nigeria; 4 Clinical Trial Consortium University of Nigeria, Nsukka, Nigeria; 5 Fakultaat III, Hochschhule Hannover University of Applied Sciences & Arts, Expo Plaza 12, 30539 Hannover, Germany; 6 Department of Physiotherapy, Faculty of Health Sciences, School of Therapeutic Studies, University of the Witwatersrand, 7 York Road, Parktown, 2193 Johannesburg, South Africa

**Keywords:** HIV, Aerobic exercise, Resistance exercise, CD^4+^ cell count, QoL, Systematic review

## Abstract

**Background:**

Compromised immune function, associated with human immune deficiency virus(HIV) infection, is improved by antiretroviral therapy(ART) which also decreases bone mineral density(BMD), and possibly the quality of life(QoL). However, physical(aerobic/resistance) exercises, were reported to induce reverse effects in uninfected individuals and were appraised in the literature for evidence of similar benefits in people living with HIV/AIDS(PLWHA). The main study objective was to evaluate the impact of physical (aerobic and resistance) exercises on CD^4+^ count, BMD and QoL in PLWHA.

**Methods:**

A systematic review was conducted using the Cochrane Collaboration protocol. Searching databases, up to June 2017, only randomized control trials investigating the effects of either aerobic, resistance or a combination of both exercise types with a control/other intervention(s) for a period of at least 4 weeks among adults living with HIV, were included. Two independent reviewers determined the eligibility of the studies. Data were extracted and risk of bias(ROB) was assessed with the Cochrane Collaboration ROB tool. Meta-analyses were conducted using random effect models using the Review Manager(RevMan) computer software.

**Results:**

Nineteen studies met inclusion criteria(*n* = 491 participants at study completion) comprising male and female with age range 22–66 years. Two meta-analyses across 13 sub-group comparisons were performed. However, there were no RCTs on the impact of physical exercises on BMD in PLWHA. The result showed no significant change in CD^4+^ count unlike a significant effect of 5.04 point(95%CI:-8.49,-3.74,*p* = 0.00001) for role activity limitation due to physical health(QoL sub-domain). Overall, the GRADE evidence for this review was of moderate quality.

**Conclusions:**

There was evidence that engaging in moderate intensity aerobic exercises (55–85% Maximum heart rate-MHR), for 30–60 min, two to five times/week for 6–24 weeks significantly improves role activity limitation due to physical health problems, otherwise physical(aerobic or/and resistance) exercises have no significant effects on CD4^+^ count and other domains of QoL. Also, there is lack of evidence on the impact of exercises on BMD in PLWHA due to the paucity of RCTs. The moderate grade evidence for this review suggests that further research may likely have an important impact on our confidence in the estimate of effects and may change the estimate.

**Electronic supplementary material:**

The online version of this article (10.1186/s12879-019-3916-4) contains supplementary material, which is available to authorized users.

## Background

Human immunodeficiency virus (HIV) infection persists as a global public health issue [1] and is presently regarded as a chronic condition [2] since the advent of Highly Active Antiretroviral therapy (HAART). This is a sequel to the significantly improved life expectancy in people living with HIV (PLWHA) who are HAART-experienced [3]. This view is given further credence by the decreasing number of HIV-related deaths post-HAART era, down from the extreme point of 2.3 million in 2005 to an estimated 1.0 million in 2016 [4]. However, high incidence of comorbidities has been recorded in PLWHA, and might be due to the high rate of metabolic abnormalities resulting from toxic side effects of antiretroviral therapy(ART) [5]. These include low bone mineral density (BMD), and consequent high risk of fracture [6], cardiovascular diseases [7]^,^ and instability of fat metabolism [8], which may be amenable to physical exercises.

Several research studies have supported the role of physical exercise as a complementary alternative therapy in the management of chronic illnesses, and apparently both aerobic and resistance exercises are beneficial to PLWHA [9].

 For instance, contemporary literature supports the conclusion that exercise has been the key strategy to improve lean body mass, cardiovascular fitness [10], improve strength [11], change mood state [5], increase BMD, reduce risk of fracture and invariably enhance quality of life (QoL) in PLWHA [12]. These developments may be attributed to the regulatory effects of structured exercise on immune function and probably as a result of expressive traction of muscles to the bones during training protocols.

Structured physical exercise at different intensities and duration has been shown to improve mental health, QoL, immune and physical function in PLWHA compared to an inactive lifestyle [3]. Aerobic exercise, when carried out for 16 weeks (30 min, 3 times/wk) at a moderate intensity, resulted in either an increased or a stable CD^4+^ cell count which improves resistance to infection [5]. In fact, Maduagwu et al. [13], reported a significant improvement in the CD^4+^ cell in a pre-test and post-test 12 week (40 min, 3 times/wk) experimental study. Similar findings were reported by Ezema et al. [14], who observed a significant increase in CD^4+^ cell count and VO_2_ max with exercise compared to the control in an 8-week (45–60 min) training program. However, Tiozzo et al. [15], reported a stable CD^4+^ cell count with exercise during a 12-week intervention (45–60 min, 3 times/week) study, unlike the control who recorded a significant decrease. Similarly, structured resistance exercise also triggers a specific immune response in PLWHA as reported by Zanetti et al. [16] who demonstrated that resistance exercise for 12 weeks (3 times/week) is effective in boosting the CD^4+^ and CD^8+^ cell counts with consequent improvement in the integrity of the immune system. Furthermore, Anandh [17] also reported an increase in the CD^4+^ cell count, marked improvement in functional capacity and positive changes in QoL in PLWHA after a 12-week progressive resistance exercise (60 min, 3times per week) intervention. Meanwhile, a significant increase in the BMD and maximum muscle strength was observed after strength training, 3 times per week, for 12 weeks [12]. However, it is by investigating the unique impact of physical exercises through relevant studies in PLWHA that its physiological and therapeutic effects on the immune system, BMD, and Qol, in this population may be determined.

Overall, there are mixed reports on whether there are therapeutic benefits associated with physical exercises on the immune system, BMD, and QoL in PLWHA. Therefore, a systematic review of the literature should be conducted to provide a synthetic knowledge that is required to guide practice. To the best of our knowledge, this has not been done and was the focus of this review. Thus, the aim of this systematic review was to determine the impact of physical exercises on immune function, BMD, and QOL in patients with HIV.

## Methods

This systematic review was certified according to the International Prospective Register of Systematic Reviews (PROSPERO) on 27 June 2017 (registration number PROSPERO 2017: CRD42017069068).

### Eligibility criteria

Eligibility criteria considered for selecting studies in the review include:

- Inclusion criteria:
  1. Type of studies
    Original research manuscripts in peer-review journals and conferences proceeding were included if published in the English Language. This design only included RCTs in the review when the following objective was evaluated: the effects of physical exercises on immune function, BMD and QoL in PLWHA.
  2. Type of participants
    The review included studies involving adult human participants aged ≥18 years. Only studies that investigated PLWHA were included, however, no specific limitation was considered with respect to the setting of the studies. The included studies were mainly carried out in clinics, health centers, hospitals or community care settings.
  3. Type of intervention
    RCTs of physical exercise (either/both aerobic and resistance exercise) intervention for PLWHA were included in the review, which was not restricted to specified dosage, form, intensity, frequency and duration of intervention or follow-up period after aerobic intervention or limited to weight training, isometric and isotonic strengthening for resistance exercise in PLWHA.
  4. Timing
    There was no specified length of the interventions or the follow-up of outcomes.
  5. Types of outcome measures
    Studies that reported changes in outcome measures of immune function (e.g. CD^4+^ count or viral load), BMD (e.g. osteoporosis or osteopenia) and QOL (e.g. physical function, the performance of social roles, emotional status, and cognitive function) in PLWHA were included in the review. Studies were included regardless of whether an outcome of interest was accounted for as a primary or secondary outcome in the first article, so far as a clear analysis was carried out for each outcome. All outcome variables were collated as they were accounted for in individual studies, and the original description in those individual studies was not modified. Clinical results, detailed by individual studies were analyzed and graded.
- Exclusion criteria:
  1. Studies without an exercise or physical activity component.
  2. Narrative review syntheses, systematic reviews, opinion papers, letters and any publication without primary data and/or explicit description of the methods.
  3. For duplicate publications from the same study, the most recent or most comprehensive publication was used.

### Information sources and search strategy

A search strategy was formulated and piloted as shown in Additional files 1, 2 and 3. This was based on the guidelines of the Cochrane Handbook for Systematic Reviews [18] and advice for Health Care Review by the Centre for Reviews and Dissemination [19]. This formulated strategy was further adapted for use in other databases. Eight databases (CINAHL, the Cochrane Library, ProQuest, AMED, MEDLINE, PubMed and Web of Science Core Collection) and trial registers and directory of open-access repository websites were searched by the reviewers - FOR and EFE - using controlled vocabularies and keywords: HIV/AIDS, Seropositive, aerobic exercises, resistance exercises, strengthen exercises, physical exercises, exercise program, exercise intervention, CD4^+^, immune function, bone mineral density, bone turnover, and QoL. Additionally, searches were performed from the reference lists of identified studies.

### Study record, selection process, and data management

Literature search results were exported into RefWorks to check for duplication of studies. Bibliographic records were exported from RefWorks into Microsoft Excel (Microsoft. Microsoft Excel. Redmond, Washington: Microsoft, 2010. Computer Software) [20] to facilitate the management and selection of articles for inclusion. Eligibility questions and forms for the screening of the studies included in the review were then developed, piloted and subsequently refined. The title, abstract and full texts of selected studies were independently screened for eligibility by F.I.O and E.F.E based on the review eligibility criteria. Differences of opinions occurring at any stage regarding inclusion or exclusion were resolved by discussion and reflection, in consultation with S.C.I.

### Quality appraisal and risk of bias

Adopting the Cochrane Collaboration Tool for Risk of Bias Assessment (Table 8.5a of the Cochrane Handbook for Systematic Reviews of Interventions) [21], risk of bias for each of the included studies were evaluated by two authors in six key domains: (i) selection bias (random sequence generation, allocation concealment); (ii) performance bias (blinding of personnel and participants); (iii) detection bias (blinding of outcome assessments); (iv) bias due to attrition (incomplete outcome data, including dropouts and withdrawals); (v) reporting bias (selective reporting) and (vi) other bias (other sources of bias not elsewhere addressed) (Additional file 4) [18].

The procedures undertaken to assess each domain for each study was explicitly described and rated as ‘high risk’ or ‘low risk’. The risk of bias in a study was reported as unclear if there were insufficient details in the original study. In such instances, the study investigators were contacted to provide the required details. The judgments for the risk of bias was made independently by the first reviewer and the same with the second reviewer, based on the criteria for judging the risk of bias (Additional file 4) [18]. Both reviewers made judgments regarding the risk of bias independent of each other. Areas of differences were resolved by discussion and reflection, or in consultation with S. C. I.

### Data item

Data were collected from variables including authors’ references, participants’ characteristics, inclusion and exclusion criteria, study sample size, components of the intervention, the intervention setting, who delivered the intervention, the duration of the intervention and follow-up (where available), attrition rate, aspects of outcome assessed, the outcome measurement, methods/techniques, results, conclusions and funding sources.

### Data synthesis and assessment of heterogeneity

The review question of the impact of physical exercises on immune function, BMD, and QOL, in patients with HIV/ AIDS, was answered. In doing this, all quantitative study outcomes which analyzed the effectiveness of these interventions were presented, considered and combined in a proof table. The Proper statistical method was used for different variables: for a continuous variable, weighted mean differences were applied when outcomes are uniform or standard mean difference when different outcomes are used with 95%: CI while for a dichotomous variable, the Risk ratio was applied with 95%: CI. This review also includes a meta-analysis to find pooled effect sizes across studies, using a random-effects model relying on the level of heterogeneity of intervention effects. Heterogeneity was assessed using the Cochrane’s χ^2^ test (10% significance level) and Higgins I^2^ for which values of 25, 50, and 75% shows low, medium and high heterogeneity respectively as stipulated by the guidance in the Cochrane Handbook for Systemic Reviews of Interventions [19].

### Data and sensitivity analysis

Investigation and presentation of outcomes were made using the primary outcome. Studies with homogenous characteristics in terms of design, intervention, and comparator(s) were pooled together for meta-analysis using a random-effects model [18]. Heterogeneous studies were interpreted by narrative synthesis following the guidelines of the Centre for Reviews and Dissemination to explore the relationship and findings between the included studies [19]. Sensitivity analysis was done to decide the impacts of studies with a high risk of bias on the general outcomes with and without these studies.

### Rating quality of evidence and strength of recommendation

The quality of evidence of the studies was evaluated to determine the strength of recommendation in the systematic review. This was judged utilizing the Grading of Recommendations Assessment Development and Evaluation (GRADE) approach [22] which comprise consistency; design; directness; precision; publication bias and study limitations. The individual study was graded as high risk of bias or low risk of bias, and then again individual evidence statement for this review was graded from ‘High Quality’ to ‘Very Low Quality’ according to the criteria (Additional file 5).

## Results

### Search result

Three different searches were carried out sequentially during the course of this study using the three-primary outcomes separately in the search strategy.

1. Immune function
  Searches of all sources found 127 citations, and after duplicate removal, 79 were potential for evaluation of which 16 publications were considered after topic and abstract screening for inclusion. When full-text screening was concluded, 13 articles met the inclusion criteria [13, 14, 16, 17, 23–31, 36] (Additional file 6. PRISMA flow Diagram).
2. Bone mineral density
  The result from all sources gave 18 citations, 6 duplicates were removed and after a full-text screening of 12 articles, none met the study inclusion criteria. (Additional file 7. PRISMA flow Diagram).
3. Quality of life
  The search strategy resulted in 251 citations, after deduplication and abstract review, 12 full citations were reviewed to determine whether they met inclusion criteria which after review, 10 studies met the inclusion criteria [17, 23, 24, 27, 32–37]. (Additional file 8. PRISMA flow Diagram).

### Reasons for exclusion

Three full-text articles retrieved (for immune function) were excluded because studies [38–40] did not assess immune function as an outcome. For the QoL, the 2 studies [41, 42] excluded were non-randomized clinical trials. For bone mineral density, none of the studies were RCT and therefore were all excluded.

### Included studies

#### Immune function

All the study that reported immune function as an outcome were all RCTs. 10 studies used aerobic exercise [13, 14, 23–30], 2 studies utilized resistance exercise [16, 17] while 1 study [31] combined both exercises as an intervention. Studies of aerobic exercise intervention included 7 studies with no exercise group [23, 25, 26], maintain normal daily activity group [13, 27, 30], and high-intensity aerobic exercise group [27, 29], while Ezema [14], Ogalha [24]^,^ and Terry [28] utilized conventional therapy, counselling, and soft stretching plus relaxation as control, respectively. Resistance exercise studies on the other arm also had no exercise group [17] and maintain normal activity group [16] while the combined study [31] had no exercise group as control (Table 1).

**Table 1:** Characteristics of Included Studies

Author, Year (Location of study)	Characteristics of participants Age(years) Gender Sample size. Retention (attrition)	Intervention. No of participants allocated (No that completed)	Duration of intervention	Control	Outcome	Measurement tool for outcome	Summary of result
Agin, 2001 (U.S.A)	28–66 Women *N* = 37 81% (19%)	Progressive Resistance Training 3 sets of 10 exercises (8–10 repetition / set) *n* = 12 (10) Whey protein (PRO) *n* = 12 (10) Combined (PRO-PRE) *n* = 13 (10)	14 weeks		QoL	MOS survey	Physical activity score significantly increased for PRE group (*p* = 0.02): general health perceptions (*p* = 0.03), vitality (*p* = 0.007)
Anandh, 2014 (India)	41.71 ± 5.73 *N* = 24 80% (20%)	Progressive Resistance Training. (10 RM 3x weekly) n = 12 (9)	12 weeks	No exercise *n* = 12 (10)	QoL Immune function	MOS-HIV survey CD4 count	Effective in increasing CD4 count (E.S = 0.09; *p* = 0.041) and QoL (*p* = 0.004)
Baigis, 2002 (USA)	NR *N* = 123 80.5% (19.5%)	Aerobic exercise (75–85% MHR) *n* = 68 (52)	15 weeks	No exercise *n* = 55 (47)	Immune status QoL	CD4 count MOS-HIV	No significant impact of exercise on CD4 count Significance on MOS-HIV overall health subscale (p = 0.02)
Ezema, 2014 (Nigeria)	22–63 NR *N* = 33 91% (9%)	Aerobic exercise (60–79% MHR) *n* = 17 (15)	8 weeks	Conventional therapy *n* = 16 (15)	Immune function	CD4 count	Increase CD4 count in the exercise group compared to control (ES = 0.7)
Farinatti, 2010 (Brazil)	45 ± 2 years NR *N* = 27 87% (13%)	Aerobic training (30 mins. of moderate intensity exercise (cycle ergometer), PWC 150); strengthening exercise-50mins (2 sets of 12 reps of 5 exercises at 60–80% 12 RM); and flexibility exercise- 10 min (2 sets of 30s at max. ROM of 8 exercises).	12 weeks	No treatment	Immune function	CD4 count	There was no significant change in the CD4-T cell counts either in the exercise group or the control group
Galantino, 2005 (Rwanda)	20–60 years *N* = 51 75% (25]	EX intended to foster strength, endurance, and cardiovascular exertion. (60–70% MHR).	8 weeks	Maintain normal activity	QoL	(MOS-HIV) and Spirituality Well-Being Scale (SWB).	Exercise training improved quality of life
Gillespie, 1997 (United State)	27–46 *N* = 23 78.3% (21.7%)	Aerobic exercise (60–80% MHR) *n* = 11(6)	12 weeks	No exercise. *n* = 12	QoL	MOS-HIV	No significant difference between exercise and control groups on MOS-HIV
Maduagwu, 2015 (Nigeria)	39.57 ± 10.13 *N* = 82 78% (22%)	Moderate intensity aerobic exercise (treadmill) 50–70% HRR *n* = 41 (32)	12 weeks	Maintain routine daily activities *n* = 41 (32)	Immune function	CD4 count	Significant improvement of CD4 count between pre-test and post-test in the experimental group (ES = 0.8)
Maharaj, 2011 (South Africa)	NR *N* = 52 50% (50%)	Aerobic exercise. (50–70% MHR) *n* = 26 (20)	12 weeks	SWD (as a placebo) n = 26 (6)	QoL	SF-36 Questionnaire	QoL significantly improved for the experimental group compared with the control. Physical component (ES = 0.3; *p* < 0.018) Mental component (ES = 0.2; *p* < 0.021)
Mkandla, 2016 (Zimbabwe)	42.2 ± 8.5 *N* = 160 40% (60%)	Progressive Resistance Exercise intervention To lower limb *n* = 80 (29)	12 weeks	Usual advice + normal activities *n* = 80 (35)	QoL	(EQ-5D) Euro quality of life-5 dimension	significantly improved (HRQOL) in the intervention when compared to the control group measured using the state of health visual analogue(p = 0.04)
Mutimura, 2008 (Rwanda)	21–50 years *N* = 100 97% (3%)	EXC include warmup (15 min) followed by 45–60 min of jogging, running, stair climbing, low-back & abdominal stabilization and strengthening exercises	24 weeks	No treatment	QoL	WHOQOL-BREF	Exercise training improved several components of QoL in HAART-treated HIV+ African subjects with body fat distribution
Ogalha, 2011 (Brazil)	43.15 ± 9.45 *N* = 70 90% (10%)	Aerobic exercise (75% MHR) *n* = 35	24 weeks	Counseling n = 35(28)	QoL Immune function	SF-36 CD4 count	Higher significance for patient in exercise group concerning general health, vitality and mental health significant improvement for CD4 (ES = 0.2; *p* = 0.001)
Perna, 1999 (USA)	36.75 ± 6.27 *N* = 43 65% (35%)	Aerobic exercise (70–80 MHR) n = 24 (18)	12 weeks	No exercise n = 19 (10)	Immune function	CD4 count	Significant increase with compliant exercises (ES = 0.9 p < 0.02), while significant decrease for non-compliant and control with a decrease of about 10%
Smith 2001 (U.S.A)	36 ± 6.6 *N* = 60 82% (18%)	Aerobic exercise training (60–80% MHR) *n* = 30 (19)	12 weeks	No exercise n = 30	Immune function	CD4 count	No significant change in CD4 cell count
Stringer 1998 (U.S.A)	36 ± 9 *N* = 34 76% (23%)	Aerobic exercise (Cycle ergometer) Moderate intensity (n = 9)	6 weeks	Maintain current level of activity n = 8 Heavy intensity Aerobic exercise *n* = 9	Immune function QoL	CD4 count A subset of QoL questionnaire validated prior HIV studies	Minimal change among the thee group Improvement occurred in both exercise training groups relative to control group.
Terry, 2006 (Brazil)	37.5 ± 8.5 *N* = 42 71% (29%)	Aerobic exercise (70–85% MHR) n = 21 (15)	12 weeks	Soft stretching and relaxation routine. *n* = 21(15)	Immune function	CD4 count	No significant change after exercise
Terry, 1999 (Brazil)	31 ± 8 *N* = 31 68% (32%)	Aerobic exercise Moderate intensity (55–60% MHR) n = 16 (10)	12 weeks	Aerobic exercise High intensity (75–85 MHR) *n* = 15 (11)	Immune function	CD4 count	No appreciable changes in the moderate or high intensity exercise group
Yar’zever, 2013 (Nigeria)	39.2 ± 12.75 yrs. *N* = 40 93% (7%)	Aerobic exercise (cycle ergometer) (50–60 MHR) *n* = 20	12 weeks	Normal daily activities *n* = 20 (17)	Immune function	CD4 count	Significant deference in CD4 count between pre and post experimental group (ES = 0.4; *p* < 0.05) and decrease viral load, while control had a decrease in CD4 count and increase viral load
Zanetti, 2016 (Brazil)	41.1 ± 10.1 N = 30 NR	Resistance exercise 3 sets of 6 exercise (6–12 RM/set) n = 15	12 weeks	Maintain daily habit n = 15	Immune function	CD4 count	Increase in CD4 count from pre- post intervention.

Key: NR = Not recorded; QoL = Quality of life; RM = Repetition maximum; SWD = Short wave diathermy; MOS = Medical outcome study, SF-36 = Short form MOS; MHR = Maximum heart rate; HRR = heart rate reserve; ES = Effect size

#### Quality of life

Studies that assessed QoL as an outcome were all RCTs. Five studies had aerobic exercise as intervention [23, 24, 27, 32, 33, 34], three studies had resistance exercise as an intervention [17, 32, 35] and two studies combined both interventions [36, 37]. Aerobic exercise and combined studies had no exercise [23, 33, 37], maintain daily activity [27, 36], and Short-wave diathermy as a placebo [34], with counselling [24] groups as control while resistance exercise studies had no exercise [17], usual advice plus normal activities [35] as control groups. One of the three studies for resistance exercise assessed the effects of co-intervention of progressive resistance exercises (PRE)-and Whey protein [32]. This study also included a comparison group of whey protein only (Table 1).

### Participants of included studies

#### Immune function

A total of 639 participants were included in this arm of the review (i.e. participant recorded at baseline). Participant were all HIV male and female adults with CD^4+^ cell count ranging from < 150 cells/mm^3^ to > 850 cells/mm^3^ with age range 22–63 years and were on a HAART regimen. Five studies had participant located in Brazil [16, 24, 28, 29, 31], one in India [17], three in Nigeria [13, 14, 30], and four in U.S. A [23, 25–27] (Table 1).

#### Quality of life

Participants (661) included in this arm of the review were both male and female PLWHA at various stages of the disease, with CD^4+^ cell count < 100 cells/mm [3] to > 1000 cells/mm [3]. The age ranged from 28 to 66 years and their location were Brazil [24], India [17], South Africa [34], U.S.A [23, 27, 32, 33,], Rwanda [36, 37] and Zimbabwe [35] (Table 1).

#### Outcome of intervention

All but four of the included studies assessed for immunological function using CD^4+^ cell count or viral load [32–34]. Ten studies assessed QoL outcome using Medical Outcomes Study (MOS) Health Survey [17, 23, 32, 33], 36-Item short-form Health survey MOS [24, 27, 34, 36], Euro quality of life-5 dimensions (EQ-5D) [35] and World Health Organization Quality of Life (WHOQOL)-BREF questionnaire [37]. Three studies assessed physical endurance [23, 26–28], but Ezema [14] and Perna [25] accessed cardiopulmonary function while Agin [32], Mkandla [35], and Zanetti [16] assessed muscle strength. Also, four studies assessed lipid profile [24, 27–29] while Zanetti [16] assessed inflammatory markers.

#### Risk of Bias in included studies

Tables 2 and 3 provides information on quality appraisal and risk of bias in the included studies. All the included studies carried out random sequence generation and were free of selective reporting bias. Four studies [13, 17, 23, 32] described the sequence for allocating participants into study groups. Four studies [17, 26, 34, 35] further reported assessor and personnel blinding and were thus judged low risk in this regard.

**Table 2:** Quality Appraisal/ Risk of Bias of included studies (Cochrane tool)

	Adequate Sequence generation		Allocation concealment		Blinding		Incomplete outcome data addressed		Selective outcome reporting		Free of other bias		Summary ROB^c^. Quality*
	Judgement	Description	Judgement	Description	Judgement	Description	Judgement	Description	Judgement	Description	Judgement	Description	
Agin 2001	Yes	Quote: “Sequential randomization was generated …” Comment: Probably done	Yes	Quote: “Group assignment was executed by the principal investigator and concealed until the time of treatment” Comment: Probably done	Yes	Comment: Outcome measurement not likely to be influenced by lack of blinding	Yes	Quote: “Adherence to exercise training was 94% for PRE” Comment: Probably done	Yes	Comment: Study outcome adequately reported.	Yes	Comment: Probably appears to be free from other sources of bias.	Low risk High Quality
Anandh 2014	Yes	Quote: “All 24 subjects after baseline assessment were randomly allotted” Comment: Probably done	Yes	Quote: “…into two groups by using sealed envelopes.” Comment: Probably done	Yes	Quote: “…physiotherapist who is blinded to group allotment” Comment: Probably done	Yes	Quote: “Intention to treat analysis for all outcome measures was carried out” Comment: Probably done	Yes	Quote: “All three outcome measures were tested at baseline and end of three months of intervention” Comment: Probably done	Yes	Comment: Probably appears to be free from other sources of bias.	Low risk High Quality
Baigis 2002	Yes	Quote: “Study identification (ID) numbers were randomized” Comment: Probably done	Yes	Quote: “…in advance and placed in sequentially numbered opaque envelopes” Comment: Probably done	Yes	Comment: Outcome measurement not likely to be influenced by lack of blinding	Yes	Quote: “Intent to treat analysis was used for the physiologic variables” Comment: Missing data imputed using appropriate method	Yes	Quote: “outcomes between the intervention and the control group were determined at baseline, 8, and 15 weeks” Comment: Probably done	Yes	Comment: Probably appears to be free from other sources of bias.	Low risk High Quality
Ezema 2014	Yes	Quote: “Eligible participants were randomized…” Comments: Probably done	No	Quote: “using simple random assignment into the exercise group and the control group respectively.” Comment: Probably not done	Yes	Comment: Outcome measurement not likely to be influenced by lack of blinding	Yes	Comment: Missing data adequately accounted for.	Yes	Comment: Study outcome recorded pre and post treatment.	Yes	Comment: Probably appears to be free from other sources of bias.	Low risk High Quality
Gillespie 1997	Yes	Quote: “a table of random numbers was used to randomly assign each individual…” Comment: Probably done	No	Comment: Probably not done	Yes	Comment: Outcome measurement not likely to be influenced by lack of blinding	Yes	Comment: Missing data reported appropriately	Yes	Quote: “… variables in the pretest to posttest changes in quality of life…” Comment: Probably done	Yes	Comment: Probably appears to be free from other sources of bias.	Low risk High Quality
Maduagwu, 2015	Yes	Quote: “This random assignment involved the authors…” Comment: Probably done	Yes	Quote: “…papers were then wrapped, placed and mixed in a basket.” Comment: probably done.	Yes	Comment: Outcome measurement not likely to be influenced by lack of blinding	Yes	Quote: “22% attrition rate was recorded general,..” Comment: probably done	Yes	Quote: “… the pre – and post – test values of the variables in the experimental and control groups.” Comment: Probably done	Yes	Comment: Probably appears to be free from other sources of bias.	Low risk High Quality
Maharaj 2011	Yes	Quote: “…then randomly assigned by means of a computer…” Comment: Probably done	No	Comment: Probably not done	Yes	Quote: “the researchers and assistants blinded to the scores…” Comment: probably done	Yes	Comment: Missing data imputed.	Yes	Quote: “questionnaire was completed by both groups on entry and monthly …” Comment: Probably done	Yes	Comment: Probably appears to be free from other sources of bias.	Low risk High Quality
Mkandla 2016	Yes	Quote: “Random allocation was applied to both participants and clinics.” Comment: Probably done.	No	Comment: Probably not done	Yes	Quote: “An assessor-blinded RCT was conducted” Comment: probably done.	Yes	Quote: “Intention to treat analysis was applied using the last observed value for missing data (60%).” Comment: Probably done	Yes	Quote: “…profile scores at baseline and post-intervention” Comment: Probably done.	Yes	Comment: Probably appears to be free from other sources of bias.	Low risk High Quality
Ogalha 2011	Yes	Quote: “70 subjects were randomized” Comment: Probably done	No	Comment: Probably not done	Yes	Comment: Outcome measurement not likely to be influenced by lack of blinding	Yes	Comments: No missing Data for the Exercise group	Yes	Comment: Study outcome adequately reported.	Yes	Comment: Probably appears to be free from other sources of bias.	Low risk High Quality
Perna 1999	Yes	Quote: “… were randomly assigned” Comment: Probably done	No	Comment: Probably not done	Yes	Comment: Outcome measurement not likely to be influenced by lack of blinding	No	Comment: Probably not done	Yes	Quote: “... as a between- subjects factor and time point (baseline and 3-month)…” Comments: Probably done.	Yes	Comment: Probably appears to be free from other sources of bias.	Low risk High Quality
Smith 2001	Yes	Quote: “subjects were randomly assigned…” Comment: Probably done.	No	Comment: Probably not done	Yes	Quote: “…except the principal investigator, were blinded to the subject’s group assignment…” Comment: Probably done.	No	Comment: Probably not done	Yes	Quote: “…variables were measured at baseline and week 12 in all subjects” Comment: Probably done	Yes	Comment: Probably appears to be free from other sources of bias.	Low risk High Quality
Stringer 1998	Yes	Quote: “… by means of a computer- generated randomization.” Comment: Probably done	No	Comment: Probably not done	Yes	Comment: Outcome measurement not likely to be influenced by lack of blinding	Yes	Quote: “Patient who dropped out of the study were uniformly distributed among the three groups.” Comment: Probably done	Yes	Comment: Study outcome adequately reported.	Yes	Comment: Probably appears to be free from other sources of bias.	Low risk High Quality
Terry 1999	Yes	Quote: “Subject were then randomized to participate…” Comment: Probably done.	No	Comment: Probably not done	Yes	Comment: Outcome measurement not likely to be influenced by lack of blinding	Yes	Comment: Missing data adequately accounted for.	Yes	Quote: “Before the program, at 6 weeks and the end of the program blood was collected…” Comment: Probably done.	Yes	Comment: Probably appears to be free from other sources of bias.	Low risk High Quality
Terry 2006	Yes	Quote: “Those who met the inclusion criteria were then randomized to participate” Comment: Probably done.	No	Comment: Probably not done	Yes	Comment: Outcome measurement not likely to be influenced by lack of blinding	Yes	Quote: “Compliance with the sessions in both groups was 100%” Comment: Probably done.	Yes	Quote: “Before and after the intervention, 12-h fasting venous blood samples were collected” Comment: Probably done	Yes	Comment: Probably appears to be free from other sources of bias.	Low risk High Quality
Yar’zever 2013	Yes	Quote: “They were randomly assigned to either…” Comment: Probably done	No	Comment: Probably not done	Yes	Comment: Outcome measurement not likely to be influenced by lack of blinding	Yes	Comment: No missing data	Yes	Quote: “…dependent variables (CD4 count and viral load) were measured before and after cycle exercise programme” Comment: Probably done.	Yes	Comment: Probably appears to be free from other sources of bias.	Low risk High Quality
Zanetti 2016	Yes	Quote: “Then, they were randomly allocated” Comment: Probably done	No	Comment: Probably not done	Yes	Comment: Outcome measurement not likely to be influenced by lack of blinding	No	Comment: Probably not done	Yes	Comment: outcome measured pre and post intervention	No	Comment: Baseline incompatibility.	High risk Low Quality

^c^ Summary for risk of bias (ROB) was assigned using the Cochrane tool for risk of bias

*Studies were subsequently rated low quality trials (having high ROB) or high quality trials (having low to moderate ROB) if there was ≥3 or < 3 identifiable sources of bias respectively (Abaraogu et al. 2017)

**Table 3:** Risk of bias in individual studies for combined Studies

Study	Sources/Potential sources of bias^a^								
	Selection bias		Performance bias	Detection bias	Bias due to attrition	Reporting bias	Other bias	Summary of bias	Quality index^b^
	Random sequence generation	Allocation conceal-ment	Participants and personnel blinding	Blinding of outcome assessment	Incomplete data	Selective reporting			
Mutimura et al. 2008	No	Yes	Yes	Yes	No	No	–	High	Low
Farinatti et al. 2010	No	Yes	No	Yes	No	No	Small sample	Low	High
Galantino et al. 2005	No	Yes	Yes	Yes	No	No	–	High	Low

The Cochrane’s tool was used to determine and summarize possible sources of risk of bias in the included studies (Cochrane 201) (Yes indicates the presence of or potential presence of a source of bias)

^a^ summary risk of bias in included studies was presented

^b^ studies were subsequently rated as low quality trials (i.e. having high risk of bias) or high quality trials (i.e. having low to moderate risk of bias if there was ≥3 or < 3 identifiable sources of bias respectively (Abaraogu et al. 2017)

Overall, 571 participants withdrew from the included studies accounting for ~ 54% withdrawal rate (571/1062 participants at baseline). Withdrawal rates within individual studies ranged from 3% [37] to 60% [35] (Table 1). However, a high risk of bias due to attrition exists as 15 of the 19 included studies (78.9%) reported withdrawal rates of > 15% while 1 study [16] did not provide information on incomplete outcome reporting. The remaining 3 studies were judged the low risk of bias due to attrition (25%) with withdrawal rates of < 15% [14, 24, 30]. The withdrawal rate between comparison groups was similar in most groups. Almost all the included studies mentioned participant who was not complying with their exercise intervention or withdrew from the study. (Table 1 shows the proportion of participants who dropped from individual studies). All Authors but one [16] reported information on adherence to the exercise intervention. Adherence rate ranged from 40 to 93% [30, 35].

#### Narrative synthesis

##### Immune function

One study [31] which assessed immune function, and which was not included for meta-analysis combined aerobic and resistance exercise among 27 HAART treated HIV-infected patients (age 45 ± 2 years). This intervention was carried out in Brazil for 12 weeks whereby the exercise group (*n* = 19) were involved in aerobic training (cycle ergometer) for 30 min of moderate intensity, strengthening exercise (2 sets of 12 repetitions of 5 exercises at 60–80% 12 Repetition Maximum) for 50 min and flexibility exercise (2 sets of 30s at maximum range of motion of 8 exercises) while the control group (*n* = 8) received no treatment. Immune function (CD^4^ and CD^4^%) were determined by specific monoclonal antibodies using fluorescein isothiocyanate and phycoerythrin monoclonal antibodies using a whole blood staining method. The study reported no significant change in the CD^4^ T-cell count in either the exercise group or the control group (*p* = 0.19 for CD^4^ T-cells and *p* = 0.22 for CD^4^%) [31].

##### Quality of life

Similarly, two studies [36, 37] which investigated QoL also employed a combined exercise approach in PLWHA. In Rwanda, Mutimura [37] recruited 100 HIV individuals (21–50 years) with body fat redistribution (BFR) that were on HAART for greater than 6 months to find out the effect of exercise training on QoL. The participants (exercise group) were involved in a 6-month supervised exercise (EXS) programme which consists of warm-up exercise followed by 45–60 min of jogging, running, stair climbing, low-back & abdominal stabilization and strengthening exercises while the control group did not undergo any treatment. The outcome of interest (i.e. QoL) was measured at baseline and after 6 months using WHOQOL-BREF (short-form instrument) evaluating physical, psychological, independence and social relationship domains. Findings from this study state that over the 6-month training period, significant improvements were observed in BFR + EXS group for the psychological, independence, social relationships (*p* < 0.001) domains of QoL compared to BFR + noEXS group [37]. The other study also with a combined intervention on functional outcomes and QoL [36] was also carried out in Rwanda with 38 PLWHA (20–60 years) on HAART greater than/ 3 months. The EXS group received 8 weeks of exercises with the goal to foster strength, endurance, and cardiovascular exertion while the control group maintained normal daily activities. QoL outcome was assessed using the Medical Outcome Short Form (MOS-HIV) and report states that there is an improvement in QoL from participants in EXS group when compared to control group in overall health perception subscale (*p* = 0.04) [36].

The above studies [36, 37] from inference have a common relationship in setting, type of intervention, study design and outcome of interest. Results from both studies are also similar suggesting a positive effect of combined aerobic and resistance exercise on PLWHA on HAART (> 3 months). The robustness of this synthesis is low and should be interpreted with caution because of the quality attributed to the studies (having a high risk of bias) judging by Cochrane risk of bias assessment tool [18].

#### Meta-analyses – effects of interventions

This review conducted two meta-analyses across thirteen sub-group comparisons which included meta-analyses for immune function (CD^4+^ cell count) and QoL. The sub-group comparisons of the meta-analyses were:

1. Aerobic exercise compared to no exercise as a control
2. Aerobic exercise compared to normal routine activity as control
3. Moderate intensity aerobic exercise compared to high-intensity aerobic exercise
4. Aerobic exercise compared to other controls (conventional, counseling and soft stretching plus relaxation)
5. Resistance exercise compared to control (no exercise and maintain normal routine activities).
6. Physical activity limitation due to health
7. Role activity limitation due to physical health
8. Bodily pain
9. General health perception/overall
10. Vitality/Energy
11. Social limitation due to physical/emotional problems
12. Role activity limitation due to emotional problems
13. Mental health.

Three of the included studies compared aerobic exercise with no exercise control group [23, 25, 26]. Three studies compared aerobic exercise with normal routine activity [13, 27, 30]. Two studies compared moderate-intensity aerobic exercise to high-intensity exercise [27, 29]. Ezema [14], Ogalha [24] and Terry [28], compared aerobic exercise with conventional therapy, Counselling, and soft stretching plus relaxation as control respectively (Table 1). Two studies compared resistance exercise to either no exercise or maintain normal activities [16, 17]. For the QoL outcome, three studies included in the review [24, 33, 34] compared aerobic exercise with control. In all domains of QoL, only role activity limitation due to physical health showed a significant difference. Meta-analyses were limited for the QoL due to the outcome reported and outcome tool for the remaining studies.

### Heterogeneity

**Fig. 1:**
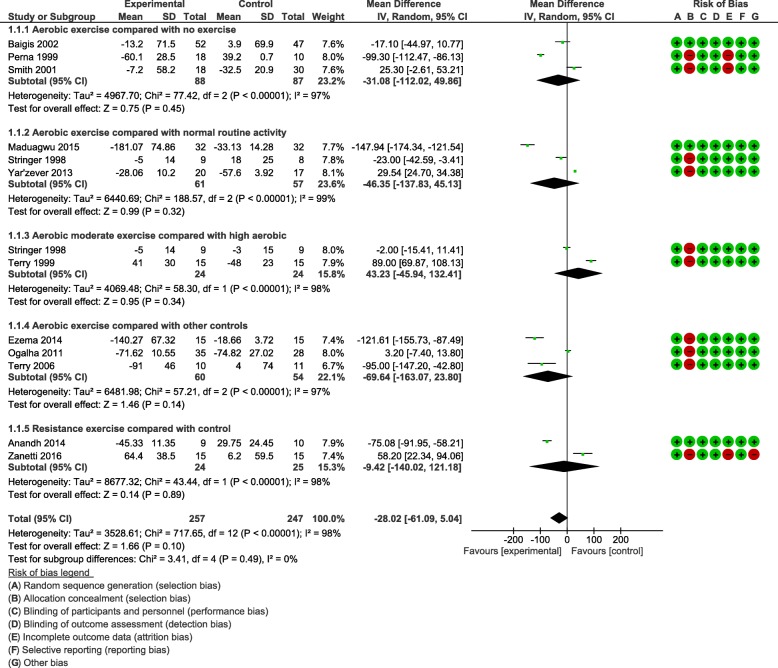
Forest plot for CD_4_^+^ cell count

**Fig. 2:**
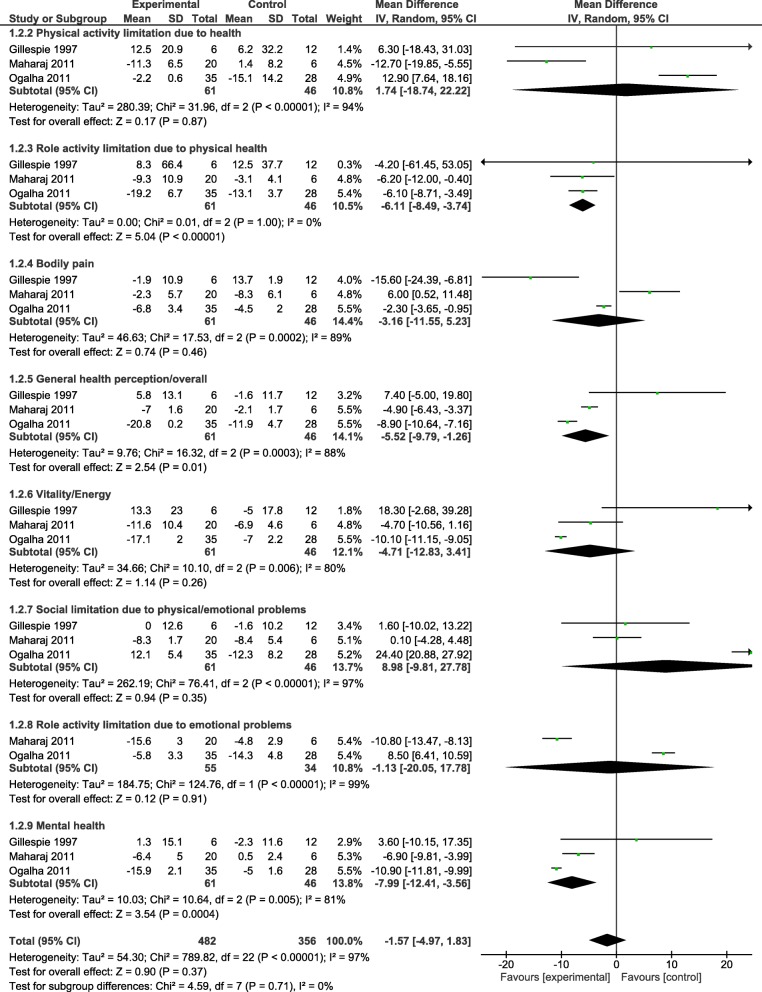
Forest plot for Quality of life

Heterogeneity (*p* < 0.1) was evident in the 2 meta-analyses which could be as a result of the differences in gender, location, and type of intervention. Sensitivity analysis was carried out with those greater than two studies since heterogeneity exists in the meta-analysis, thus the results and reasons include:

I. **Immune function (CD** ^**4+**^ **count cell)**
  Most of the nineteen included studies assessed CD^4+^ cell count as an immunological outcome. Five sub-group meta-analyses were performed for CD^4+^ cell count. All the analyses demonstrated no statistically significant change in the CD^4+^ cell count between comparison groups Weighted Mean Difference: − 28.02 cells/mm^3^, 95% CI: -61.09, 5.04, *p* = 0.10) (forest plot- Fig. 1). Results demonstrated a non-significant trend towards an increase in CD^4+^ cell for participants in the aerobic exercise compared to no exercise; aerobic exercise compared to normal routine activity; moderate intensity aerobic compared to high-intensity aerobic exercise; aerobic exercise compared to other controls and resistance exercise compared to control. The results of the meta-analyses indicated no difference in the CD^4+^ cell count for aerobic exercise compared to no exercise; aerobic exercise compared to normal routine activity; moderate intensity aerobic compared to high intensity aerobic; aerobic exercise compared to other controls. Likewise, no difference in CD^4+^ cell count was found for resistance exercise compared to control.
II. Heterogeneity – CD^4+^ count
  All the sub-group meta-analyses were statistically significant for heterogeneity (*p* < 0.1). Sensitivity analyses did not indicate a change in the overall effects of exercise on CD^4+^ cell count.
III. Quality of life
  Ten of the nineteen included studies assessed QoL. Meta-analyses were performed for three studies [24, 33, 34] on 8 sub-domains for QoL. The result of meta-analyses showed no statistically significant difference in all but one sub-domain (role activity limitation due to physical health) (forest plot- Fig. 2). This represents a clinically important improvement in role activity limitation due to physical health compared to control (no exercise, placebo, and counseling).
IV. Heterogeneity – QoL
  All but one sub-group meta-analyses were statistically significant for heterogeneity (p < 0.1). Sensitivity analyses indicated the same overall effect of exercise on QoL.
V. Grade rating
  The effect estimate demonstrated the overall significant effect of 5.04 points (95% CI: -8.49, − 3.74, *p* < 0.00001) for role activity limitation due to physical health (Qol sub-domain) when comparing aerobic exercise group to controls, which can be accepted as moderate evidence. The true effect is likely to be close to the estimate of the effect, but there is a possibility that it may be substantially different. This outcome was downgraded from high to moderate GRADE quality of evidence, because of the inability of authors to conceal allocation in the assignment of participants to experimental and control groups.

## Discussion

Meta-analyses for immune function (CD^4+^ cell count) showed that neither aerobic exercise nor resistance exercise intervention carried out for a period of 8–24 weeks had an impact on CD^4+^ cell count of PLWHA. The results also showed that neither aerobic exercise nor resistance exercise is safe for medically stable adults living with HIV/AIDS. This finding is based on few available reports/literature on the impact of either aerobic or resistance exercise on CD^4+^ cell count in HIV patients and also on participants that finished their exercise protocol prescription and for whom adequate follow-up data were provided.

Most of the studies that assessed CD^4+^ cell count were included in a meta-analysis [13, 14, 16, 17, 23–30, ]. Generally, evaluating either aerobic exercise or resistance exercise with controls among PLWHA suggests minute to no impact on CD^4+^ cell count. This agrees with the conclusion from previous reviews by Jaggers & Hand [5]^,^ Nixon [10], and O’Brien [43, 44]. Aside from these meta-analyses, individual studies that assessed CD^4+^ cell count reported increase in CD^4+^ cell count of effect size (Cohen’s *d*) 0.9, 0.7, 0.8, 0.2, 0.9, 0.4, 1.2 [13, 14, 16, 17, 24, 25, 30] respectively, suggesting a mean/pooled (0.73) large effect of both aerobic and resistance exercise on HIV patient while the remaining studies reported minimal or no change in CD^4+^ cell count [23, 26–29, 31]. Among the Ten studies that assessed the QoL as an outcome, Meta-analyses conducted for three studies [24, 33, 34] suggest that aerobic exercise only demonstrated significant improvement in one sub-domain of QoL assessment (role activity limitation due to physical health). These findings give credence to an earlier review by O’Brien [44] that the QoL improved for physical function, role emotional, and mental health sub-domains among exercisers compared to non-exercisers.

Individual studies’ results show that progressive resistance exercise group (PREG) tends to record improved physical activity score, general health perceptions, and vitality [32]. PREG group improved in QoL assessed by MOS scales [17, 23] and another using health visual analog [35]. Aerobic exercise also significantly improved the QoL (physical and mental component; general health, vitality, and mental health) of the experimental group compared to the control [24, 34] respectively. Likewise, Stringer [27] reported an improvement for both moderate-intensity aerobic exercise and heavy intensity aerobic exercise when compared to the control group while combined intervention (aerobic and resistance) improved several components of QoL [36, 37]. In contrast, Gillespie et al. [33] reported no improvement between exercise and control group using MOS-HIV scale.

### Bone mineral density

Evaluation of the impact of physical exercise on BMD was not included in this review due to the paucity of RCTs in PLWHA, assessing BMD as an outcome.

### Quality of evidence

Since the interventions in this review were aerobic exercise and resistance exercise, the possibility of blinding participants and exercise supervisors, was not feasible. The exact impact of the absence of blinding on the extent and bearing of the treatment impact is unclear, and however, may constitute a high risk of performance bias [45–49]. On the other hand, some outcomes were self-reported, which also prompted a high innate risk of detection bias when blinding of members was unrealistic. The researchers chose not to downgrade studies for this ‘risk of bias’ item alone. However, reasons for allotting studies a high risk of bias were not due to lack of blinding. Furthermore, the GRADE evidence of this review was of moderate quality suggesting that further research may likely have an important impact on our confidence in the estimate of effect and may change the estimate.

## Conclusions

### Implication for practice

Engaging in moderate intensity aerobic exercises (55–85% Maximum heart rate- MHR), for 30–60 min, two to five times per week for 6–24 weeks can lead to significant improvement in role activity limitation due to physical health problems (a sub-domain of QoL), otherwise physical (aerobic or/and resistance) exercises have no significant effects on immune function (CD^4+^ cell count) and other domains of QoL. However, lack of RCTs on the effects of physical exercises on the BMD in PLWHA makes it difficult to reach a scientific conclusion that will guide practice. The findings from this study are very important because a previous study [50] has shown that functional limitations of PLWHA affect their care needs and ability to perform social roles such as employment. According to Crystal et al. [50], the limitation in complex roles among PLWHA – such as working at a job, working around the house, or going to school - was more prevalent than limitation in most specific physical tasks. Among physical tasks, limitation was more prevalent in energy-demanding activities including climbing stairs (43%) or walking > 1 block (26%) than in self-care tasks such as bathing and dressing (14%). Moreover, symptom intensity, pain, and fatigue were strongly associated with these limitations. Therefore, the evidence from this study recommends physical exercises (specifically moderate intensity aerobic exercises) as an effective clinical tool for addressing the wellbeing of PLWHA such that improved management of these disease symptoms using physical exercises might improve physical and social functioning at no extra financial cost, and with little or no side effects to the PLWHA.

### Implication for research

The research found few studies on the impact of physical exercises (i.e. aerobic or resistance exercise or the combination of both exercise types) on immune function, and QoL for adults living with HIV, but none on the BMD. Moreover, the few studies identified are also of relatively low quality. All these reasons cumulate and emphasize the need for further research studies in this area. The lack of RCTs on the impact of physical exercises on BMD in PLWHA is an important gap that needs to be addressed in future studies. The increasing reports that bone demineralisation is amenable to physical exercises in non-HIV infected population [51] highlights the possibility of the translational relevance of such studies in HIV/HAART-experienced PLWHA considering the fact that HIV/HAART are associated with bone loss. The study population included both younger and older adults, as well as both male and female participants without considering the variation in the age of participants which could possibly influence the results. The small number of participants per study, that completed the exercise intervention (*n* = 6–52) suggest that the included studies may have lacked sufficient power to detect treatment effect or that the sample size is smaller than what would be required to detect a clinically important benefit. Therefore, further studies should address the limited number of RCTs and the weaknesses of the available studies as already mentioned above, otherwise it will be difficult to reach a scientific conclusion that will guide practice on the effects of exercises on such parameters as CD4^+^ cell count, BMD, and other domains of the QoL.

### Limitation of study

The findings of this review might be limited for the following reasons: i) This review included a small number of studies that actually met the study criteria, Meta-analyses conducted involved a maximum of 3 studies and had a range of 24–88 participants in each sub-group. In addition, the generalizability of the results may be limited by the fact that the general findings among individuals who complied with the exercise prescriptions might not reflect the genuine experience of exercise among adults living with HIV/AIDS. However, since all the studies were RCTs, it should be expected that the studied population, should to a reasonable extent, be representative of the exercise experiences of most PLLWHA. Thus, it affords us sufficient confidence to conclude that the estimate of evidence provided in these studies should approximate the real-life experiences of most of the PLWHA. Nevertheless, the inability to conduct meta-analyses for all the included studies that assessed for QoL due to limited reporting of outcome variables, should also be acknowledged as a limitation.

## Additional files

Additional file 1: Search strategy in PubMed for immune function. The *MESH* terms used to search the Pubmed database for evidence of the impact of physical exercises on immune function in HIV conditions. (DOCX 15 kb)

Additional file 2: Search strategy in PubMed for Bone Mineral Density. The *MESH* terms used to search the Pubmed database for evidence of the impact of physical exercises on Bone Mineral Density in HIV conditions. (DOCX 15 kb)

Additional file 3: Search strategy in PubMed for Bone Mineral Density. The *MESH* terms used to search the Pubmed database for evidence of the impact of physical exercises on Quality of Life in HIV conditions. (DOCX 15 kb)

Additional file 4: The Cochrane Collaboration’s tool for assessing the risk of bias (adapted from Table 8.5a in the Cochrane Handbook for Systematic Reviews of Interventions). The tool used for assessing the risk of bias in the selected studies from the database. (DOCX 35 kb)

Additional file 5: Quality of Evidence and Definitions (adapted from Guyatt et al., 2008). The weighing factors that define the quality of evidence for each of the selected studies. (DOCX 17 kb)

Additional file 6: PRISMA checklists for immune function. A diagrammatic flow of how the studies on the impact of exercise on immune function were selected from the database considering the stated eligibility criteria. (DOCX 43 kb)

Additional file 7: PRISMA checklists for Bone mineral density. A diagrammatic flow of how the studies on the impact of exercise Bone mineral density were selected from the database considering the stated eligibility criteria. (DOCX 39 kb)

Additional file 8: PRISMA checklists for quality of life**.** A diagrammatic flow of how the studies on the impact of exercise quality of life were selected from the database considering the stated eligibility criteria. (DOCX 44 kb)

## Abbreviations

AIDS: Acquired immune deficiency syndrome
AMED: Allied and Complementary Medicine Database
ART: Highly Active Antiretroviral therapy
BFR: body fat redistribution
BMD: Bone mineral density
CINAHL: Cumulative Index to Nursing and Allied Health Literature
CON: Control
EMBASE: Excerpta Medica database
EQ-5D: Euro quality of life-5 dimensions
ES: Effect size
EXS: Exercise
GRADE: Grading of Recommendations Assessment Development and Evaluation
HIV: Human immune deficiency virus
HRR: Heart rate reserve
MeSH: Medical subject heading
MHR: Maximum heart rate
MOS: Medical Outcomes Study
NHREC: Nigeria Health Research Ethics Committee
NR: Not recorded
PEDro: Physiotherapy Evidence Database
PLWHA: People living with HIV/AIDS
PREG: progressive resistance exercise group
PRG: Progressive resistance exercise group
PRISMA: Preferred Reporting Items for Systematic Reviews and Meta-analyses
PROSPERO: International Prospective Register of Systematic Reviews
QoL: Quality of life
RCTS: Randomized control trials
RevMan: Review Manager
RM: Repetition maximum
ROB: Risk of bias
SF-36: Short form MOS
SWD: Short wave diathermy
WHOQOL: World Health Organization Quality of Life
WHS: Women’s Health Study
