# Supplementary material for: Impact of physical exercises on immune function, bone mineral density, and quality of life in people living with HIV/AIDS: a systematic review with meta-analysis
Source: BMC Infect Dis. 2019 Apr 24;19:340. doi: 10.1186/s12879-019-3916-4 (PMC6480814; doi:10.1186/s12879-019-3916-4)
Supplement: Supplementary file 2 — Search strategy in PubMed for Bone Mineral Density. The MESH terms used to search the Pubmed database for evidence of the impact of physical exercises on Bone Mineral Density in HIV conditions. (DOCX 15 kb) [file 12879_2019_3916_MOESM2_ESM.docx]

**Additional file 2**

| **CONCEPT** | **SEARCH TERMS** |
| --- | --- |
| **Population** | 1. HIV 2. AIDS 3. Human immunodeficiency virus 4. HIV infection 5. Acquired immunodeficiency syndrome, 6. Retrovirus 7. Retroviridae 8. 1 OR 2 OR 3 OR 4 OR 5 OR 6 OR 7 |
| **Intervention** | 1. Exercise 2. Exercise training 3. Aerobic exercise 4. Resistance exercise 5. Resistance training 6. Weightlifting 7. Isometric exercise 8. Strength training 9. 9 OR 10 OR 11 OR 12 OR 13 OR 14 OR 15 OR 16 |
| **Study design** | 1. Randomised controlled trial 2. Clinical trials 3. Random allocation 4. Control groups 5. 18 OR 19 OR 20 OR 21 |
| **Outcome** | 1. Immune function 2. CD4 cell count 3. viral load 4. 23 OR 24 OR 25 5. 8 AND 17 AND 22 AND 26 |
