# Supplementary material for: Impact of physical exercises on immune function, bone mineral density, and quality of life in people living with HIV/AIDS: a systematic review with meta-analysis
Source: BMC Infect Dis. 2019 Apr 24;19:340. doi: 10.1186/s12879-019-3916-4 (PMC6480814; doi:10.1186/s12879-019-3916-4)
Supplement: Supplementary file 5 — Quality of Evidence and Definitions (adapted from Guyatt et al., 2008). The weighing factors that define the quality of evidence for each of the selected studies. (DOCX 17 kb) [file 12879_2019_3916_MOESM5_ESM.docx]

Additional file 5

| **High Quality** | Evidence derived from many studies of high quality such that further research is very unlikely to change our confidence in the estimate of effect |
| --- | --- |
| **Moderate Quality** | Evidence derived from most studies of moderate quality and/or few high-quality studies such that further research is likely to have an important impact on our confidence in the estimate of effect and may change the estimate |
| **Low Quality** | Evidence derived from mostly low-quality studies and/or few studies of moderate quality such that further research is very likely to have an important impact on our confidence in the estimate of effect and is likely to change the estimate |
| **Very Low Quality** | All evidence derived from studies of low-quality studies such that any estimate of effect is very uncertain |
