# Supplementary material for: Impact of physical exercises on immune function, bone mineral density, and quality of life in people living with HIV/AIDS: a systematic review with meta-analysis
Source: BMC Infect Dis. 2019 Apr 24;19:340. doi: 10.1186/s12879-019-3916-4 (PMC6480814; doi:10.1186/s12879-019-3916-4)
Supplement: Supplementary file 7 — PRISMA checklists for Bone mineral density. A diagrammatic flow of how the studies on the impact of exercise Bone mineral density were selected from the database considering the stated eligibility criteria. (DOCX 39 kb) [file 12879_2019_3916_MOESM7_ESM.docx]

Additional file 7

Records identified through database searching
(n =18)

Identification

Records after duplicates removed
(n =12)

Screening

Records screened
(n =12)

Records excluded
(n =11)

Full-text articles assessed
for eligibility
(n =1)

Eligibility

Full-text articles excluded,
with reasons
(n =1)

1= Combined aerobic and resistance exercise

Studies included in
qualitative synthesis
(n =0)
