# Supplementary material for: Impact of physical exercises on immune function, bone mineral density, and quality of life in people living with HIV/AIDS: a systematic review with meta-analysis
Source: BMC Infect Dis. 2019 Apr 24;19:340. doi: 10.1186/s12879-019-3916-4 (PMC6480814; doi:10.1186/s12879-019-3916-4)
Supplement: Supplementary file 8 — PRISMA checklists for quality of life. A diagrammatic flow of how the studies on the impact of exercise quality of life were selected from the database considering the stated eligibility criteria. (DOCX 44 kb) [file 12879_2019_3916_MOESM8_ESM.docx]

Additional file 8

Records identified through database searching
(n=251)

Identification

Records after duplicates removed
(n =160)

Screening

Records screened
(n =160)

Records excluded
(n =148)

Full-text articles assessed
for eligibility
(n =12)

Full-text articles excluded,
with reasons
(n =4)

2= Not RCT

2= combined aerobic and resistance exercise

Eligibility

Studies included in
qualitative synthesis
(n =8)

Included

Studies included in
quantitative synthesis
(meta-analysis)
(n = 3)
